# Acknowledgement to reviewers

**DOI:** 10.1530/ETJ-12-A1

**Published:** 2023-02-01

The editorial board wishes to thank the following individuals who have helped to review manuscripts for *European Thyroid Journal* during 2022. Their assistance is gratefully acknowledged.

A Abbara

R Abgral

P Abraham

C Agathe-Azencott

K Agrawal

N Agrawal

S Ahmadi

R Ajjan

T Akamizu

E K Alexander

M Almquist

C Alvarez

A S Alzahrani

F Amiri

S Andersen

S L Andersen

T E Angell

E Angelucci

A Antonelli

A Antoniou

A Aranda

A Arora

P Asfar

B Asvold

F Azizi

M Azizi

K Badenhoop

J H Baek

A Baranowska-Bik

S Bardet

J C Barrett

L Bartalena

F Basolo

D Bassett

S Bath

A Bauer

A Beckers

T Bednarczuk

A Ben Hamou

C Benbassat

F N Bennedbaek

I M Bensenor

B Berger

A C Bianco

B Biondi

R P M Biscolla

P Bisschop

A Blanchard

S Bliddal

K Boelaert

A Boelen

J Boguslawska

J Bojunga

M Bongiovanni

B Bongue

S J Bonnema

M G Borrello

F Borson-Chazot

G A Brent

G Brenta

G Brigante

J Brito

K Brix

T Brix

T Brue

D Buchholz

C Buffet

T Busso

A Butler

M E Cabanillas

B Cakir

I Campi

S Cannegieter

U Canpolat

A R Cappola

A Carle

A Carre

D P Carvalho

B Casey

J Cassereau

M G Castagna

P Caturegli

F Ceriotti

N Chabbert-buffet

P Chanson

T D Cheetham

C E Cherella

L Chiovato

I Christakis

M Christ-crain

S Christin-maitre

D S Cooper

B Corvilain

S Costagliola

G J Cote

R Coutant

P K Cramon

E Crivicich

L Crotti

Z Culig

F A Cunha

R Czepczynski

A F Daly

V Darras

F D’Aurizio

L Davies

M De Felice

E de Koster

M Dentice

A Derakhshan

R Dhillon Smith

B Di Jeso

J W Dietrich

C Dinauer

H Dobnig

P J Dolman

C Dosiou

P M Doubilet

M S Draman

A Dumitrescu

L Duntas

C Durante

C J Eastman

A Ebrahimi

A Eckstein

M Edmunds

G Effraimidis

L Elbers

R Elisei

C Eloy

M F Erdogan

M Erichsen

M Eszlinger

D G Ezra

J A Fagin

U Feldt-Rasmussen

M Fichna

N Fichter

S Filetti

M Finessi

F Flamant

E Fliers

K Frank-Raue

F Frasca

A Frasoldati

M Frates

J D French

L Fugazzola

D Fuhrer-Sakel

F Fulciniti

R F Gagel

J C Galofré

G Gerrard

G Gets

S Ghai

C Giani

F Gibb

M Gild

B Giometto

T Giordano

L Giovanella

R Glonek

A R Glover

B Goichot

I G Goodfellow

B Goswami

M Gotthardt

G Grani

A Greco

J E Greenlee

R K Grewal

L Groussin Rouiller

L G Grunnet

M Gurnell

A Gwanyanya

A G Hall

D Halsall

D Hartl

T Hasebe

M Hashibe

M Haymart

A Heald

L Hegedus

J V Hennessey

A Hernandez

D Hernando

H Heuer

C Higham

O Hilmi

S N Hinkle

C Hintschich

J K Hoang

S Hyer

P Iglesias

F Illouz

T Ittermann

L Iughetti

H Iwasaki

Y Iwatani

B Jarzab

S Jiang

J Jiskra

J Jonklaas

S Joustra

C Juhlin

Y S Jung

G J Kahaly

P Kamenicky

E Kandil

E Kebebew

N Kelderman-Bolk

R Kelly-Laubscher

J Kero

D-H Kim

W G Kim

C M Kitahara

N Kiyota

J Koehrle

K Kohagura

T Korevaar

G Krause

M Kremer

A Kus

A Kyriacou

C La Vecchia

J Lado-Abeal

A G Lai

L Lane

B Lang

J Langer

V Langlois

M Lantz

G Lanzolla

F Latrofa

S Leboulleux

S Lee

L Leenhardt

J Lefrandt

J Leger

F Lesueur

A M Leung

A Lewinski

M Ligtenberg

J Lim-Dunham

T P Links

M J Livhits

C Llorens-Cortes

A Lonardo

N López-Alcántara

J Lorch

L Lorente-Poch

D Lucini

Y Luo

M Luster

S Lytton

J Maciel

A L Maia

W Makis

G Malfatto

J Mammen

M Marino

K Markou

P Marques

T Martin

E T Massolt

G Mauri

S Mayerl

C McCabe

A McKeon

D McLeod

D S A McLeod

M Medici

D Mekahli

M Melo

P L Meroni

L Min

G Mintziori

L Miranda-Alves

A Miranda-Filho

A Mitchell

J Mittag

R Mogulkoc

E Molinaro

H Monpeyssen

C F Mooij

C Moran

R Moreno-Reyes

D Morganstein

I Muller

A Muth

M Muzza

D G Na

S Nagala

E B Nagy

E V Nagy

S Nakamura

K-H Nam

R Negro

K Newbold

M Niedziela

K Nirantharankumar

I Nixon

I J Nixon

J E Noel

D Nostrand

C Nucera

M T Nunes

A M O’Carroll

M Oczko-Wojciechowska

K Okamura

O E Okosieme

J Orgiazzi

M Papaleontiou

E Papini

Y-G Park

R Paschke

E N Pearce

S H S Pearce

R P Peeters

G Pellegriti

G B Perego

R Pereira

M Perez Lopez

P Perros

C Peters

A Piccardo

A Piekielko-Witkowska

F Pitoia

T Planck

T S Plantinga

J Pohlenz

M Polak

K A Ponto

R K E Poortvliet

Y Popov

K Poppe

K G Poppe

M L Prasad

M Proietti

M Puig-Domingo

T Rago

M Ralli

M Rauner

V Raverot

M Rayman

S Razvi

S Refetoff

J L Reverter

C Rhee

I J S Ribeiro

M Ries

M Rodolfo

P W Rosario

M Roseland

E Rossi

M Rotondi

M Ruchala

G Russ

N Russell

M Ryder

M M Sabra

L M Sachs

F Saito

M C Salerno

M Sales-Sanz

B Salvatore

M Salvi

F Santini

A M Sawka

M Schlumberger

N Schoenmakers

L Schomburg

R E Schweppe

E J S Sherman

Y B Shi

L Sigurd

R Simo

J Simões-Pereira

E Simsek

P Singh

N Singh Ospina

R S Sippel

G Sitoris

M Skovsager Andersen

R C Smallridge

P Soares

T Solymosi

S Sorrenti

J Sosa

C Spitzweg

A Stagnaro-Green

S Steinhubl

O Stich

D Stoian

A Stoupa

M Strachan

T Struja

G P Sykiotis

G Szinnai

D Taieb

K-i Takayama

K Takeshima

H M Targovnik

P N Taylor

Y Tenenbaum-Rakover

W Teng

F N Tessler

A Teumer

P Thuillier

A Todica

D Toft

Y Tomer

A Toniolo

D J Topliss

B Torlinska

K Toulis

G Treglia

P Trimboli

D Tumino

M Tuncel

M Tuttle

D Unuane

B Vaidya

F Vaisman

M Vaisman

R Valcavi

C van Kinschot

A S P van Trotsenburg

M Vanderpump

M-C Vantyghem

F A Verburg

D Verity

F Viazzi

D Villagelin

D Viola

W E Visser

P Vitti

L Vogt

J Wadsley

S G Waguespack

S Wakino

G Walls

L Wartofsky

Y Watanabe

T Watt

A Weghofer

V Wenter

W M Wiersinga

F Williams

C Winther

E Wirth

L Wirth

E Witkowska-Sedek

B A Woodruff

M Xing

C-J Xu

S Yamashita

D Ylli

J S Yoon

H Zaidi

M C Zatelli

M Zimmermann

